# Problematizing content moderation by social media platforms and its impact on digital harm reduction

**DOI:** 10.1186/s12954-024-01104-9

**Published:** 2024-11-09

**Authors:** André Belchior Gomes, Aysel Sultan

**Affiliations:** 1 Release Legal Emergency & Drugs Service, London, UK; 2 Technical University of Munich, Munich, Germany; 3 Monash University, Melbourne, Australia

**Keywords:** Digital drug markets, Harm reduction, Illicit drugs, Instagram, Social media, TikTok

## Abstract

Recent years have marked a shift in selling and buying illicit psychoactive drugs from darknet cryptomarkets to publicly accessible social media and messaging platforms. As more users turn to procuring drugs this way, the role of digital harm reduction has become particularly urgent. However, one of the main obstacles complicating the implementation of digital harm reduction is the increasingly automated content moderation by the social media platforms. While some platforms are less restrictive about harm reduction content (e.g., TikTok), others implement higher degrees of moderation, including the removal of individual content and banning of entire profile pages (e.g., Instagram). This article discusses community guidelines of five popular social media and messaging platforms and their content moderation tools. It aims to highlight how these guidelines may be inadvertently curbing the dissemination of harm reduction and health promotion materials, and erroneously interpreting it as a promotion of drug use and sales. The discussion concludes that digital harm reduction requires transdisciplinary collaboration of professional organizations, researchers, and social media platforms to ensure reliable implementation of digital harm reduction, and help build safer digital communities.

## Introduction

As selling and buying illicit psychoactive drugs has become more prevalent on social media and messaging apps (hereafter social media for brevity), digitalization and adaptation of harm reduction to these rapidly evolving platforms is in increasing demand. However, the topic of digital harm reduction remains under-studied and has not been given due consideration in the international drug policy landscape. The emerging digital drug market studies of the past decade have all highlighted the importance of self-organized and community-led harm reduction measures. These were investigated in terms of determining the purity and quality of drugs [1, 54], and the internet itself as a space for sharing safer drug use techniques and community building. A key feature making digital harm reduction particularly useful includes the protection of anonymity afforded by digital technologies [19, 49]. While anonymity has enabled bottom-up harm reduction through user forums, other features especially prominent in cryptomarkets include product and vendor review systems and more sophisticated operations such as automatic escrow services [44]. Early drug cryptomarket studies underscored how people who use drugs saw the value in discussing drugs online, be it for personal advice, safer use practices or for general exchanges, yet also noted the legal challenges of discussing illicit drugs online [6] leading to shutdown of platforms and dissolving communities for safer use knowledge. However, the question of how user exchanges and relevant harm reduction strategies could better adapt to public social media platforms, and how these platforms in turn could impact such efforts, require more attention.

Broadly defined, harm reduction is a pragmatic set of strategies that aim to reduce harms associated with drug use both in terms of immediate intervention as well as preventive support for continued or habitual use [42, 56]. Over the years, definitions of harm reduction evolved and adapted to broad policy measures and intervention programs, ranging from harm reduction as public health strategy and alternative to criminalization [43], to recognizing pleasures of drug use, peer safety and care [53], to harm reduction as a liberatory practice and a political philosophy [35]. Despite the different uses of the term, legal and social punitive environments have remained primary structural enablers of drug use related harms (e.g., [58]). Due to such barriers, harm reduction strategies often pursue making safe*r* drug use knowledge to be the primary goal of any harm reduction strategy. Harm reduction is not only an established set of practical interventions to reduce harms but is also a public health framework that reduces structural and systemic barriers that impede people from accessing health care services and achieving the highest attainable standard of physical and mental health [48].

Traditionally, harm reduction was oriented at injecting users of so-called ‘hard drugs’ and people who live with chronic infectious diseases, while recreational drug use had been of lesser priority. However, harm reduction oriented at recreational users has acquired a special importance since the emergence of digital drug markets [7], which made the social supply of drugs easier [16]. Given the ubiquity of social media apps, the use of these platforms to sell and buy drugs has invoked the interest of researchers and harm reduction professionals alike [17, 25]**.** Studies have shown that recreational (non-dependent) young users are the primary demographic of sellers and buyers of drugs on social media [4, 50, 65]. This is likely because most drugs sold on social media are the so-called ‘party drugs’ used in nightlife settings and music festivals [68] and cognitive enhancement or the so-called ‘study drugs’ [21]. In addition, the COVID-19 pandemic accelerated buying drugs online [18, 31], especially by young people experiencing increased mental health challenges [9]. More users reported buying in larger quantities than usual and buying drugs online for the first time [51], noting easy access as one facilitator [25, 65]. Social networks, mediated through social media, have a strong potential to spread health-promoting behaviors and messaging – although these have traditionally been abstinence-based, recovery-oriented messages [59]. Against this backdrop, recent research studies underscore the potential for harm reduction on social media [66].

In this article, we are interested in digitally adapted harm reduction that can be communicated via social media platforms to disseminate educational as well as health intervention and warning materials. In this context, we suggest that adaptation of harm reduction to digital platforms invites *hybridity* to its definition. This is because social media platforms and their automated content moderation tools complicate distinguishing between different forms of harm reduction. This is primarily due to the fact that internet platforms flatten hierarchies of knowledge and can thereby reshape intended purposes and participation of target audiences.

A critical obstacle, however, is that harm reduction content is frequently erased from social media platforms: companies are equating harm reduction strategies – whether these are information on health-related concerns, best practices to reduce harms, or simply an ad-hoc advice on safer use – to the promotion of drug use or even engagement in criminal activities. The removal of this content is justified by content moderators who follow specific community guidelines, which operate in a quasi-legislative form that allow or prohibit certain content on a particular platform. The inadvertent result of harm reduction content being banned from platforms has significant consequences not only for those running harm reduction pages, but for the potential audiences that could benefit from their content.

## Understanding community guidelines and content moderation

Content moderation is a key quality regulation of online spaces; it is the main mechanism through which social media platforms examine content against their terms of services and community guidelines to determine its suitability. Community guidelines are determined by terms of service that provide a legal framework. The tool that is more impactful for harm reduction content is the community guidelines; these act as guiding principles with varying degrees of justifications for the appropriateness of content. As Gillespie writes, [27] community guidelines “constitute a gesture”; they demonstrate platforms’ willingness to uphold legislation of the state they operate within. These often translate into upholding certain principles such as freedom of speech or condemnation of abusive language, and ultimately exemplify how they are able to govern themselves without public oversight.

The process of content moderation is becoming highly automated, with algorithms increasingly playing a key role in detecting and managing user-created content. These developments to personalize individually consumed content blur the boundaries between professional and peer-led knowledge. Among other issues, this raises the question of what harm reduction means in a digital space, and how it should adapt to the continuously changing landscape of social media. Social media platforms are governed by the private demands of the enterprises that are responsible for their community of users; there is a considerable challenge in moderating a transnational community composed of a diverse set of interests, and based in countries with at-times diverging sets of laws and customs. Researchers have explored how social media companies can be considered reluctant “custodians” of the internet due to the uneasy task of moderating various forms of online communication [27, 52]. While routine moderation ensures adhering to community guidelines by removing ‘problematic’ content, it is sometimes a cat-and-mouse game with users looking to circumvent being moderated and finding creative ways to avoid censure [26, 28].

Social media companies (e.g., Instagram, TikTok, Snapchat) manage these digital spaces and control their access, exercising significant power through their platform’s terms of services and community guidelines. In this role, social media companies’ global teams of content moderators, assisted by technology “serve as setters of norms, interpreters of laws, arbiters of taste, adjudicators of disputes, and enforcers of whatever rules they choose to establish” [27]: 5). Due to the transnational nature of social media companies, they tend to have a ‘universal’ approach to how they apply their community guidelines,to avoid public scrutiny or business disruption, they often err on the side of caution when moderating content, removing content that could be seen as breaking their established guidelines. This can potentially erase the cultural differences, and set narrow standards for what could be considered normal or offensive. From the perspective of harm reduction, which is better developed in some parts of the world, but with certain measures can be seen quite inappropriate in others, is a great example of that (e.g., [64]). Rather than develop community guidelines that are adapted to each and every jurisdiction within which they operate, which would come at a huge cost, it is easier to develop ‘universal’ guidelines and implement them thusly (see also [57]). As Common [15] has described efficiency in content moderation is often preferred over a case-by-case approach. The resultant effect is that certain discussions or activities are more susceptible to being moderated and subsequently prohibited due to a stringent interpretation of community guidelines.

Social media companies often take unilateral and opaque decisions to ban social media accounts, communities and groups that are disseminating harm reduction content. For example, in 2018, Facebook deleted ‘Sesh Safety’, a group of 50,000 people which primarily shared harm reduction advice between people, and which had an internal moderation process to delete any drug buying or selling within the group. Then-Facebook (now Meta) declined to comment on why the group had been deleted [38]. The Loop, a British harm reduction organization legally accredited to test drugs and share harm reduction messages by the British Government, has also had their content deleted on social media platforms.^1^ Several accounts posting on intersectional content discussing race and the use of psychedelics were banned from Instagram in 2022, with no response from Meta when their owners prompted the company for justification [33]. Established news outlet DoubleBlind had its Facebook and TikTok posts deleted repeatedly in the past, they also stated that their account on Instagram was shadow-banned multiple times restricting other posting features, too [34]. Sudden account deletion is also frequent for psychedelic-related businesses, journalists or online personalities,losing their accounts can have serious financial consequences, particularly for those whose livelihood is dependent on social media communications [41].

Other communities have also struggled with similarly stringent interpretation of guidelines: scholars examining social media discussions on self-harm have highlighted that moderation – as a form of censure – may be silencing positive discourses around mental health and create barriers to accessing much-needed support resources [70]. With machine-learning algorithms increasingly used to parse through thousands of social media posts, detecting community guidelines or copyright infringements, banned words, images or other content [30, 69], the broad-brush practices leading to overlooking important content is likely to become even more complicated. Coupled with the understanding that machine-learning algorithms can reproduce existing human biases of the people training them (e.g., [10, 57]), it is likely that social media platforms will continue to ban harm reduction and other content unabatedly.

While certain social media profiles dedicated to digital harm reduction have managed to accrue significant followings, the fact that they are often moderated in an opaque manner, and even banned from participating in social media calls for a nuanced understanding into how content moderation policies are co-shaping the digital ecosystem of drug-related information, including harm reduction content. Hence, the aim of this discussion article is not merely to argue that social media community guidelines are restricting harm reduction content in a prohibitive fashion, but to *emphasize the complexity behind such tools and illustrate the problematization of crucial content*. To this end, the following sections draw on examples of specific community guidelines and tools such as shadow-banning. However, it is beyond the scope of this article to investigate the broader impact of how social media companies shape the use of digital public sphere for participation, health promotion and safety measures – a particularly pertinent issue for the bottom-up character of drug harm reduction. What follows is not an exhaustive list of all the tools used to moderate drug-related content; the purpose is rather to prompt discussions and further research ideas into the consequences of banning such content.

## Tools and examples

To better contextualize the impact of content moderation on harm reduction materials, we looked at the publicly available community guidelines of five different social media platforms: TikTok, Meta (Facebook and Instagram), Snapchat and Telegram. We chose these platforms based on the reviewed literature that identifies them as the most used platforms for selling and buying drugs online [22, 50, 67]. The search included adult community guidelines, whereas guidelines for minors’ use of social media platforms were excluded. During the writing and revision of this article, different companies had altered their community guidelines multiple times. Therefore, the discussion provided below is based on the latest iterations as identified by the lead author.

## Tool 1: Community guidelines

### Meta (Facebook and Instagram)

**Fig. 1:**
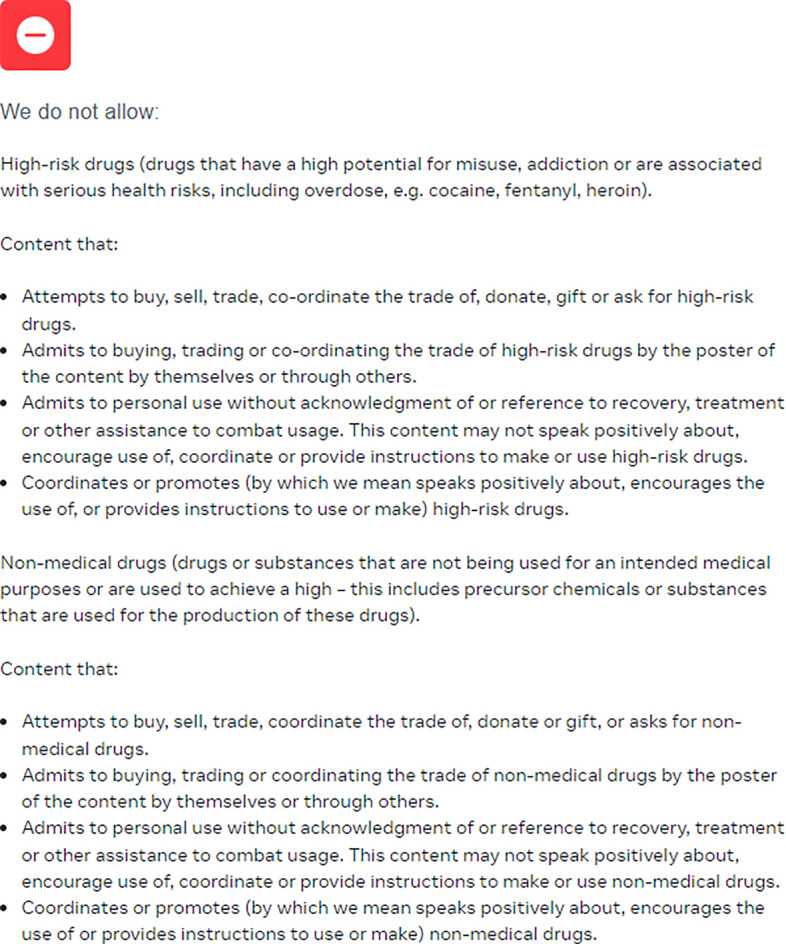
Meta’s community standards’ references to drugs and pharmaceuticals

Meta, with 2.9 billion monthly Facebook users and approximately 600 million monthly Instagram users have a harmonized content moderation policy across both social media platforms. As seen below in Fig. 1, while Meta states that they allow for “advocating for changes to regulations of goods” to happen on its platform, it explicitly does not allow for content that “admits to personal use without acknowledgment of… assistance to combat usage”^2^:

### Snapchat

**Fig. 2:**
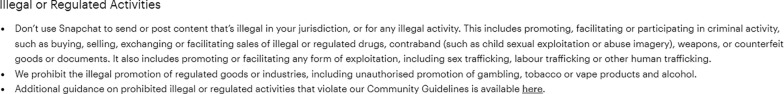
Snapchat guidelines on “Illegal or Regulated Activities”

Snapchat’s community guidelines (see Fig. 2) are brief in drug-related comments. It specifies that people should not use Snapchat to “send or post content that’s illegal in your jurisdiction, or for any illegal activity”. This includes “buying, selling, exchanging or facilitating sales of illegal or regulated drugs.” 3

### Telegram

**Fig. 3:**
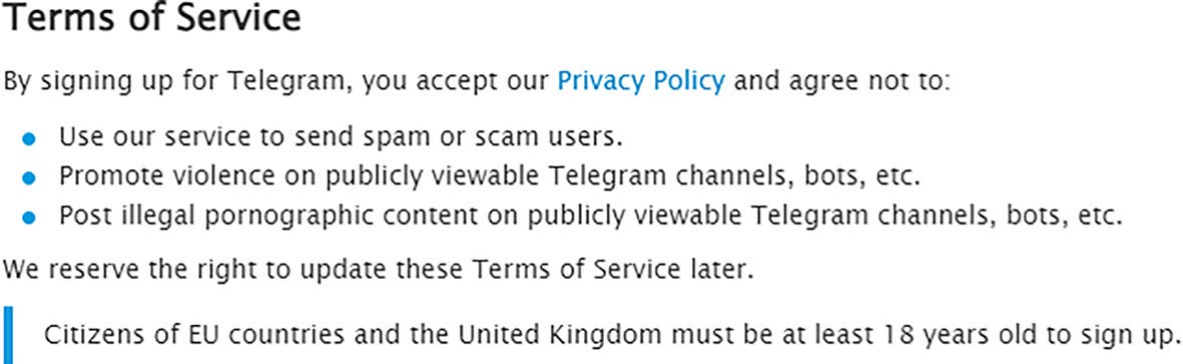
Telegram’s “Terms of Service”

Telegram has been examined as one social media app that is most adequate for purchasing and selling drugs, most likely due to its high level of data encryption and anonymity [23]. However, there is a relative paucity of evidence on how harm reduction content is disseminated and moderated on its platform (see Fig. 3. Telegram has no community guidelines; this is because users can organize themselves in groups, with their own guidelines. The overarching community guidelines that Telegram users must comply with are minimal: it states that users cannot use Telegram for scamming others, or post “illegal pornographic content”. There is no explicit mention of “drugs” anywhere in their community guidelines.^4^

### TikTok

**Fig. 4:**
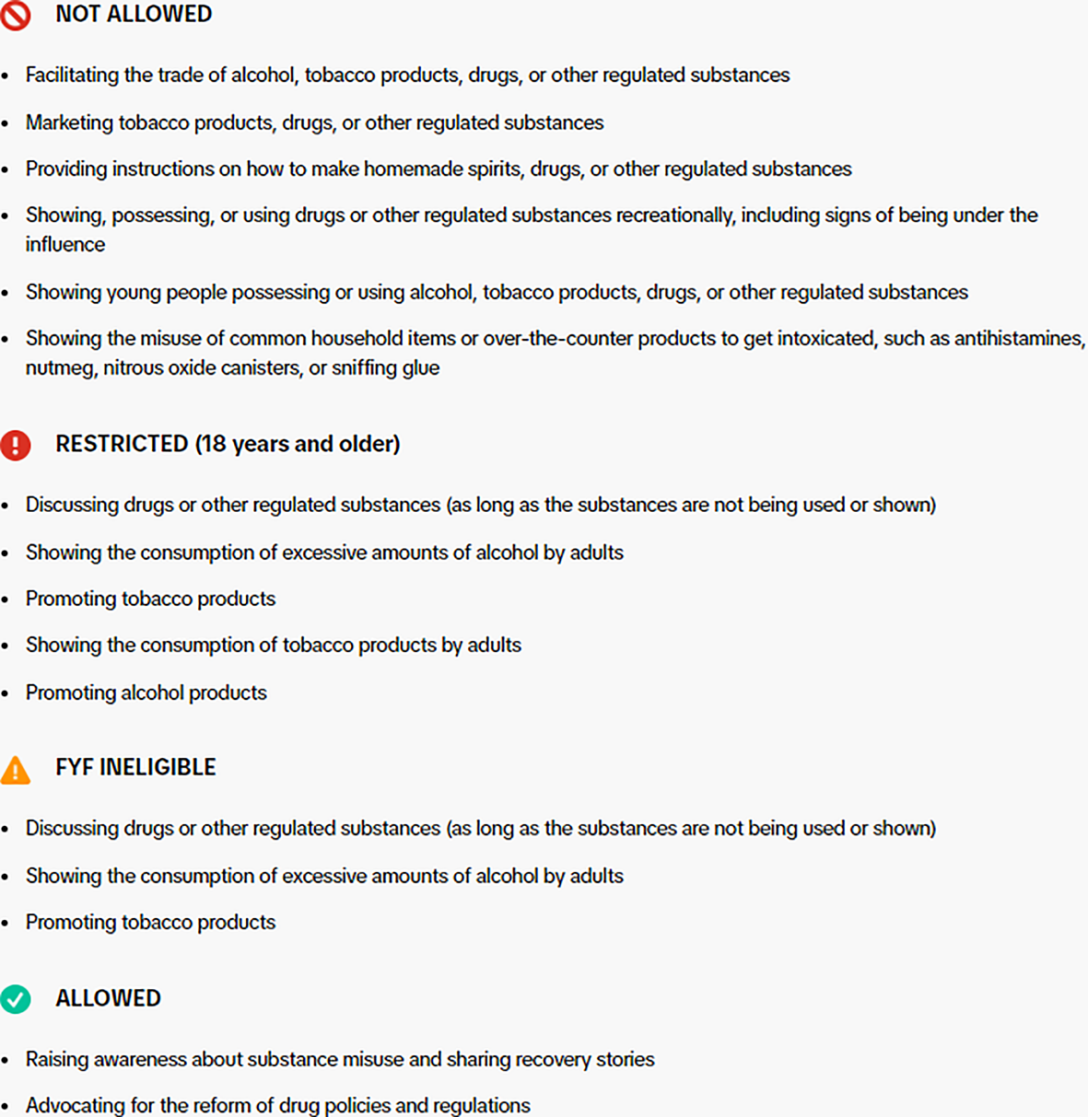
TikTok guidelines on “Regulated Goods and Commercial Activities” distinguishes drug policy reform from promoting drug use

TikTok, used by roughly one billion monthly users worldwide, also bans “showing, possessing or using drugs”. Here, TikTok limits the reach of content discussing drugs by placing an age restriction on content, as well as removing it from the “For You” Frontpage (see Fig. 4)—the main page of TikTok where global content is shown, as well as the main source of new viewers for any user. However, it allows for content “[a]dvocating for the reform of drug policies and regulations” and raising awareness of drug addiction and recovery stories.^5^

While the guidelines themselves are not explicitly prohibiting drug use related harm reduction content, it could be interpreted that a restrictive interpretation of such material (based on the observation of other platforms’ practices) would lead to its moderation or banning, particularly if content moderators are encouraged to take less risks around drug-related content [57]. This has two main effects. *Firstly*, it indirectly prevents the presence of harm reduction on social media platforms. For example, if both visual and written content is removed, then there is no possible dissemination of safer use practices. Advocating for drug policy reform would inevitably involve discussing alternative relationships with drugs that would go beyond what is currently allowed on some platforms. In other words, the passable content would be severely limited if depictions of drugs or their use is seen as promoting use.

*Secondly*, this restrictive interpretation prioritizes relationship with drugs that are based on abstinence-based and recovery content, consolidating the image that only *one* kind of relationship with drugs is acceptable. Russell [60] elaborates on how abstinence-based and recovery-oriented content is highly disseminated on social media platforms like Instagram and TikTok. However, to the best of our knowledge, there are no studies demonstrating similar social media dynamics for harm reduction content, suggesting that such content is less likely to be disseminated on the same platforms.

## Tool 2: Shadow-banning

Yet other digital mechanisms on social media platforms work against other efforts to legitimize harm reduction messages. To this end, credibility ensuring digital features, such as account verification, are often used to guarantee the veracity of content shared by accounts. The process of attributing verification is an opaque one and is rarely attributed to drug policy reform organizations. On the contrary, harm reduction-related online profiles are often shadow-banned, having their reach significantly curtailed, with their content not being recommended to other users [3]. While motivations for content moderation processes are difficult to discern from the outside, it is believed that shadow-banning is prevalent for drug-related content; users creating content with drug-related keywords can trigger flagging mechanisms that may automatically obscure results without the original poster in the known [20]. This is evident when searching for “drugs” on Instagram, where results are blocked, first displaying a message containing a mix of legal cautions and alarmist health promotion messages.^6^.

**Fig. 5:**
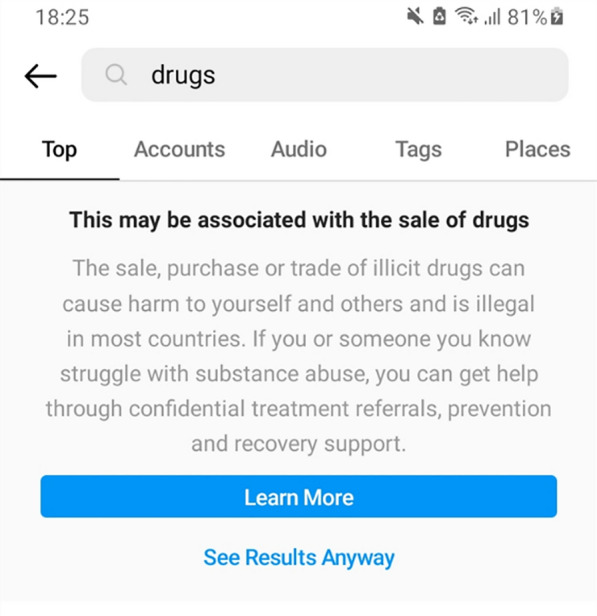
Warning message displayed by Instagram when searching for the term “drugs”. Selecting the “Learn More” button opens the government operated “Talk to FRANK” (www.talktofrank.com) webpage. These messages are prioritized over displaying search results

Figure 5 (see above) shows that selecting “Learn More” will redirect users to the British Government’s “Talk to FRANK” platform [39] – a government-funded anti-drug education campaign with sources questioning the reliability and accuracy of information [29]. Recent research to this end highlights MDMA-related content on TikTok showing that banning keywords is not always an effective strategy to prevent drug-related discussions [67]. The same study also highlights social media’s potential to disseminate harm reduction content to more vulnerable populations that may use drugs with little knowledge of potential health risks.

**Fig. 6:**
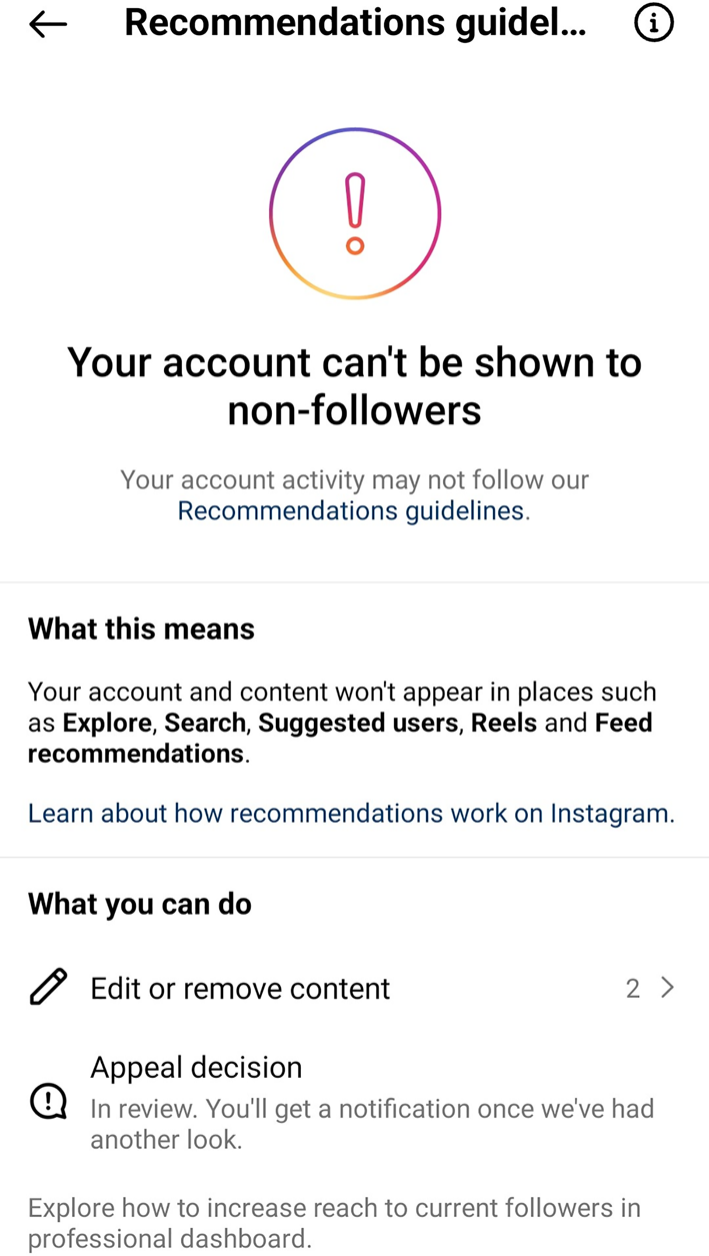
Instagram’s recommendation guidelines showing that the account run by 'Release' will not be discoverable or recommended to other users

Figure 6 (see above) demonstrates shadow-banning in action that *Release*, the drug advocacy charity, received when altering the account’s title to include the word “drugs”. This change seems to have triggered an automatic keyword review, meaning the whole account was reviewed, flagging three other pieces of older content that now needed to be edited to ensure the account is shown to other Instagram users.^7^

These features are a reflection of the complex environment of social media, one that harm reduction organizations must learn to navigate. To ensure their compliance with national legislations, social media platforms often replicate the views of national governments within which they operate. Social media companies ensure they are left alone to their own moderation practices if they can prove they are compliant with local laws and customs. However, these guidelines also aim to keep the platforms ‘clean’ for profit-driven reasons such as ensuring continued advertisement on their platforms [57]. For example, what the ‘Talk to FRANK’ collaboration seems to demonstrate is that social media companies also engage in partnerships with public institutions, perhaps to highlight their collaborative goodwill, and to ensure they comply with general norms and values. It is within social media companies’ interest to be responsive to the local governments’ demands. This was confirmed in the wake of electioneering suspicions from foreign partners in the 2020 American presidential elections, which prompted then-Facebook to invite industry and government partners to participate in self-regulation [36]. This points towards a conclusion that social media companies often use a blanket-approach to content moderation, this not only impacts vital content dissemination, but creates a complicated, opaque and lengthy process of appeal to challenge such decisions. Recent debates in social drug studies also highlight the increased use of artificial intelligence (AI) tools by alcohol and other drug industries and corporate lobbying to influence public debates and political activity [61]. The potential to leverage digital infrastructure in favor of harm reduction is yet to be researched in-depth and will require the participation of diverse stakeholders.

While the censoring of drug educational content on social media is nothing new, the fact that it is being wrongly censored when it is complying with community guidelines means such content is not sustainable on these platforms. The concern is that the internet is a crucial medium for digital harm reduction, providing networks of social support for isolated groups of drug users [12], and raising awareness around the marginalization of people who use drugs. It is also a key space to improve public awareness of existing harm reduction interventions, such as drug checking or needle and syringe programs [47]. For many users worldwide, where public access to drug-related information is non-existent or even criminalized [19], the internet is the sole location to discuss, develop and improve drug using practices. This can subvert the dominant system of drug prohibition that refuses to acknowledge controlled and pleasurable drug use and public education about safe*r* use.

There have been various community efforts to circumvent keyword flagging: the most prominent is the use of coded language. Language’s interaction with algorithm-based content moderation is known as “algospeak”: discourse used to allude to a specific topic while complying with content restrictions [63]. Emojis, however, are more context dependent in their interpretation, and thus can challenge existing content moderation practices [46]. While it is not a recent phenomenon that the internet has impacted the socio-cultural evolution of language, algospeak is particularly interesting as it has traditionally been understood to be used by groups evading moderation, such as groups spreading extremist views, misinformation or engaging in harassment or hate speech. Increasingly, algospeak is understood as a form of resistance to what is perceived as “unjust” content moderation,however, it also adds a further masking layer to the content compromising clarity. This can deteriorate the quality of the information looking to be transmitted (if it must be masked in such language that the original message is lost), or it becomes too difficult for the intended audience to find the content [63].

## Conclusion

Community guidelines are decided exogenously to the communities they impact and are applied with a particularly opaque review process where decisions may be arbitrarily reversed – or not [52]. While this process remains non-participatory, actors within social media–like harm reduction organizations and others working in similar areas–sshave the future of their accounts resting on automated and broad-brush censure. Restrictive interpretations of community guidelines by human moderators, who may be from a diverging cultural and geographical content to the original content producers [57], may mean harm reduction content is deleted, banned, or extinguished in a heavy-handed implementation of platform rules. Online drug using communities are incredibly wealthy source of information: they identify novel psychoactive substances faster than supranational specialist bodies [8], provide a forum for discussion of safer consumption practices [55], as well as disseminate harm reduction advice and potentially life-saving warnings [11].

Whether intended or not, the elimination of this content means social media companies are stifling open conversations about drug use and related harms by restricting the spread of evidence-based harm reduction information. Moreover, while governments and alcohol and other drug industry players are better able to exert influence on what content is allowed on these platforms, similar processes of influence for civil society organizations to use are not clear. This indicates that there is a technical way to better delineate the differences between different types of drug-related content: harm reduction content could be differentiated from “drug promoting” or “drug selling” content, for example. While some platforms have stated their interest in increasing transparency in their moderation practices, the opacity of moderation overall would have to be transformed. The automation of content moderation presents a serious risk to the existence of self-organized and awareness-raising communities. Not only does automated decision-making compromise human agency, it can also replicate societal biases [2, 10] such as stigmatizing attitudes towards drugs, and even exacerbate social harms, particularly when applied in a blanket manner.

Digital harm reduction organizations are reliant on their clients, and although keeping up with the ever-changing norms and trends in social media communication is laborious, they are able to develop a hierarchy of priorities, which is open to political influence from governments, and among other stakeholders, alcohol and other drug industry partners [61]. Particularly in countries where harm reduction is a legally recognized form of health intervention, governments and drug policy reform organizations, alongside drug user networks, unions and harm reduction advocates should create synergies with social media companies. It is imperative to include the voices of all named stakeholders when designing content moderation policies that affect safety and health promotion. This could ensure safe and clear communication, and controlled dissemination of reliable information. More transdisciplinary engagements between drug policy experts, researchers, and harm reduction professionals are needed to fulfill social media’s potential in preventing life-threatening outcomes and making drug use a safe*r* practice. Crucially, conversations on how social media should best operate need active involvement of the said companies themselves: targeted influence will be needed to argue that harm reduction is not only compliant with the law, but that it contributes to public safety and education. This is especially pertinent amidst ongoing legalization and decriminalization processes in some parts of the world, where there is a visible growth of online spaces for commercial products [5, 24]. This momentum can be effectively utilized to develop effective harm reduction content that will reach the target audiences without restrictions. This way we can ensure that technology becomes more equitable and reflect the ongoing changes within societal attitudes towards drugs.

Examples outlined in this discussion article underscore the need for transdisciplinary engagements to foster closer cooperation between drug policy experts, researchers, harm reduction specialists and social media companies. Research needs to ask how users are exposed to and engage with online harm reduction content to better adapt traditional harm reduction measures to online platforms and their varied features. It is also important to understand how the very medium of social media, along with the business imperatives of the companies that own them, may co-shape the discourse of online policing and behavior moderation and what their implications might be when translating some of those experiences and information to daily practices.

## Data Availability

No datasets were generated or analysed during the current study.

## Footnotes

1 Personal correspondence.

2 Access to full guidelines on Meta: https://transparency.meta.com/en-gb/policies/community-standards/regulated-goods/?source=https%3A%2F%2Fwww.facebook.com%2Fcommunitystandards%2Fregulated_goods.

3 Access to full guidelines on Snapchat: https://values.snap.com/privacy/transparency/community-guidelines?lang=en-GB.

4 Access to full guidelines on Telegram: https://telegram.org/tos/de?setln=en.

5 Access to full guidelines on TikTok: https://www.tiktok.com/community-guidelines/en/regulated-commercial-activities/.

6 Shadow-banning is the practice or process of blocking (permanently or for a period of time) a user account that has shared content that is deemed to be infringing a platform’s community standards, effectively limiting their content’s reach. Usually, the ban is not apparent to the ‘offending’ account.

7 Personal observation of the lead author who is affiliated with *Release*.
